# Validation of a counseling guide for adherence to antiretroviral therapy using implementation science*

**DOI:** 10.1590/1518-8345.3117.3228

**Published:** 2020-02-03

**Authors:** Flor Yesenia Musayón-Oblitas, Cesar Paul Cárcamo, Sarah Gimbel, Juan Ignacio Echevarria Zarate, Ana Beatriz Graña Espinoza

**Affiliations:** 1 Universidad Peruana Cayetano Heredia, Facultad de Enfermería, Lima, LIM, Peru.; 2 Universidad Peruana Cayetano Heredia, Facultad de Salud Pública y Administración “Carlos Vidal Layseca”, Lima, LIM, Peru.; 3 University of Washington, Department of Global Health, Washington, WA, United States of America.; 4 Universidad Peruana Cayetano Heredia, Instituto de Medicina Tropical “Alexander von Humboldt, Lima”, LIM, Peru.; 5 Hospital Cayetano Heredia, Estrategia Sanitaria Nacional de Prevención y Control de Infecciones de Transmisión Sexual, VIH y SIDA, Lima, LIM, Peru.

**Keywords:** Counseling, HIV, Antiretroviral Therapy, Highly Active, Alcohol Drinking, Nursing, Nursing Care, Aconselhamento, HIV, Terapia Antirretroviral de Alta Atividade, Consumo de Bebidas Alcoólicas, Enfermagem, Cuidados de Enfermagem, Consejería, VIH, Terapia Antirretroviral Altamente Activa, Consumo de Bebidas Alcohólicas, Enfermería, Cuidado de Enfermería

## Abstract

**Objective:**

to determine the contents that must be included in the usual counseling to improve the adherence to antiretroviral therapy (ART) of HIV patients, according to their different levels of alcohol consumption, and to determine the validity of the Counseling Guide in improving the adherence to ART in patients who consume alcohol using Implementation Science.

**Method:**

this is an observational study with formative and validation phases. The formative phase defined the content, approach and structure of the counseling. Validation included focus groups with patients and nurses, trial process by an expert and a pilot test. The criteria evaluated based on Implementation Science were: intervention source, evidence strength and quality, relative advantage, and complexity. The following criteria were also evaluated: usefulness, practicality, acceptability, sustainability, effectiveness; content consistency and congruence; procedural compliance and difficulties, and time spent in counseling.

**Results:**

the strength of evidence of the counseling is High-IIA, with strong level of recommendation and presenting usefulness, practicality, acceptability, sustainability and effectiveness. Eight in 11 experts argued that the Guide is clear, consistent and congruent. Initial counseling takes around 24 minutes; and follow-up counseling, 21. The instruments of the Guide present reliability levels between good and high (0.65 ≥ alpha ≤ 0.92).

**Conclusion:**

the Counseling Guide is valid to improve the adherence to antiretroviral therapy in patients who consume alcohol.

## Abstract (pt)

**Objetivo:**

determinar os conteúdos que devem ser incluídos no aconselhamento habitual para melhorar a adesão ao TARV de pacientes com HIV, conforme seus diferentes níveis de consumo de álcool, e determinar a validade do Guia de Aconselhamento para melhorar a adesão ao TARV em pacientes que consomem álcool, usando a Ciência da Implementação.

**Método:**

estudo observacional com fase formativa e de validação. A fase formativa permitiu definir o conteúdo, a abordagem e a estrutura do aconselhamento. A validação incluiu grupos focais com pacientes e enfermeiras, processo de julgamento de especialista e teste piloto. Estes foram os critérios avaliados com base na Ciência da Implementação: fonte de intervenção, força e qualidade da evidência, vantagem relativa e complexidade. Foram avaliados ainda: utilidade, praticidade, aceitabilidade, sustentabilidade, efetividade; consistência e congruência do conteúdo; cumprimento, dificuldades do procedimento e tempo empregado no aconselhamento.

**Resultados:**

o aconselhamento tem força de evidência Alta -IIA, forte nível de recomendação, apresenta utilidade, praticidade, aceitabilidade, sustentabilidade e efetividade. Oito de 11 especialistas argumentaram que o Guia é claro, consistente e congruente. O aconselhamento de início leva em torno de 24 minutos; e o de acompanhamento, 21. Os instrumentos do Guia têm um nível de confiabilidade entre bom e alto (0,65 ≥ alfa ≤ 0,92). Conclusão: o guia de aconselhamento é válido para melhorar a adesão ao tratamento antirretroviral em pacientes que consomem álcool.

## Abstract (es)

**Objetivo:**

determinar los contenidos necesarios a incluir a la consejería habitual para mejorar la adherencia al TARV de pacientes con VIH según sus diferentes niveles de consumo de alcohol, y determinar la validez de la Guía de Consejería para mejorar la adherencia al TARV en paciente que consumen alcohol usando Ciencia de la Implementación.

**Método:**

estudio Observacional con fase formativa y de validación. La fase formativa permitió definir el contenido, enfoque y estructura de la consejería. La validación incluyó grupos focales con pacientes y enfermeras, juicio experto y prueba piloto. Los criterios evaluados en base a la Ciencia de la Implementación fueron: fuente de intervención, fuerza y calidad de la evidencia, ventaja relativa y complejidad. También se evaluó: utilidad, practicidad, aceptabilidad, sostenibilidad, efectividad; consistencia y congruencia del contenido; cumplimiento, dificultades del procedimiento y tiempo empleado en la consejería.

**Resultados:**

la consejería tiene fuerza de evidencia Alta -IIA, fuerte nivel de recomendación, presenta utilidad, practicidad, aceptabilidad, sostenibilidad y efectividad. Ocho de 11 expertos, sostuvieron que la Guía es clara, consistente y congruente. La consejería de inicio toma en promedio 24 minutos y 21 minutos la de seguimiento. Los instrumentos de la Guía tienen un nivel de fiabilidad entre bueno y alto (0,65 ≥ alfa ≤ 0,92).

**Conclusión:**

la guía de consejería es válida para mejorar la adherencia al tratamiento antirretroviral en pacientes que consumen alcohol.

## Introduction

Proposing, developing and implementing effective health intervention programs aimed at specific population groups in different contexts and with defined purposes requires an evidence-based and stipulated operational milestone^(^ 1 ^)^. 

Around 2009, some scientists began to study the processes, domains and structures linked to the successful outcome of implementation, consolidating the reference framework of implementation science^(^ 2 ^-^ 4 ^)^. Implementation science facilitates the integral analysis required to implement effective interventions in a health service^(^ 2 ^)^, registering five fundamental domains for such: the intervention, the internal front (e.g.: organization), the external front (e.g.: regulations), the individuals involved and the processes^(^ 2 ^-^ 4 ^)^. 

Some experiences of implementing programs aimed at PLWHA - people living with the human immunodeficiency virus (HIV)/Acquired Immunodeficiency Syndrome (AIDS) - based on implementation science present promising results^(^ 5 ^-^ 6 ^)^; even more if a strict effort between research, service and public policies is established, including evidence patterns, participation of health providers, local government and some educational components^(^ 7 ^)^. However, no studies on the use of Implementation Science as a strategy to develop new health approach interventions and models in the country where this study was conducted were found.

Adherence to antiretroviral therapy (ART) is a crucial conduct when one wants to achieve some level of success in the effectiveness of treatment; however, access is not the sole requirement to incorporate ART, knowledge^(^ 12 ^)^ and determination are also required. However, it is often difficult to maintain an adherent behavior for a long time. Counseling is a strategy applied in different contexts and environments to empower the patient into making decisions that contribute to improve, maintain and help him care for his health^(^ 13 ^-^ 14 ^)^; to create alternatives and suggest strategies to achieve results^(^ 15 ^)^, and in this case, to maintain the adherence to treatment. 

Counseling is offered to every patient before and after the HIV diagnosis, before the onset of ART and throughout life. Several initiatives and proposals exist to provide counseling, considering different approaches and even different methods^(^ 16 ^-^ 20 ^)^; however, a scientifically validated counseling guide is unavailable in this field, as well as one that allows stating - with some level of certainty - that such counseling is effective for its application intent.

Hazardous alcohol consumption is associated with reduced adherence and reduced viral suppression^(^ 21 ^-^ 22 ^)^, it is thus necessary that these types of interventions include strategies that allow patients to have healthy behaviors and reduce alcohol consumption to improve adherence and quality of life^(^ 23 ^)^. According to the current technical standard of the selected health service, alcohol consumption patterns must be explored during the nursing interview protocol to “define nursing diagnoses or lead patients to propose their own solutions”^(^ 24 ^)^. 

Studies show that counseling is effective in adherence to ART^(^ 20 ^,^ 25 ^-^ 27 ^)^ but its methods or procedures vary; moreover, the constant turnover of nursing professionals can result in different approaches and different levels of effectiveness. Thus, a Counseling Guide that contributes to improving the adherence to ART and to reducing the levels of hazardous alcohol consumption must be developed and validated.

The objective of this study was to determine the contents that must be included in the usual counseling to improve the adherence to ART of HIV patients, according to their different levels of alcohol consumption, and to determine the validity of the Counseling Guide in improving the adherence to ART in patients who consume alcohol using Implementation Science.

## Method

The process was developed in two phases. The first phase comprised a formative research to consolidate the intervention and, since the counseling guide is aimed at people living with HIV who consume alcohol, this phase also defined the differentiated content according to the consumption levels of patients. The second phase was validation.

The Consolidated Framework for Implementation Research (CFIR)^(^ 2 ^)^ was used as a guide to establish the evaluation constructs. The domains and constructs of CFIR are available at: http://www.cfirguide.org/constructs.html. The domain “Intervention Characteristics” was used, which includes the following constructs: “intervention source, evidence strength and quality, relative advantage, and complexity”; to which the following were added: usefulness, practicality, acceptability, sustainability and effectiveness.

The formative research phase for the development of the Counseling Guide as an intervention to improve the adherence to ART in HIV patients who consume alcohol was based on the search for available scientific evidence. The “strength and quality of evidence” was evaluated. One of the results of this first phase was the elaboration of a systematic review that found that face-to-face counseling presents strong scientific evidence to be chosen as an intervention^(^ 26 ^)^. All contents of the Guide were defined in this phase.

The Guide included guidelines for its use, objectives, definition, counselor’s profile, theories in which it is based, types of counseling, counseling moments, approximate duration of each session, and procedure according to the type of counseling. All content was described in the document named “Counseling Guide to improve the adherence to therapy in patients with HIV and who consume Alcohol”.

The Guide was developed for validation in the hospital of a capital in a South American country. It is a hospital of an integrated health network that serves approximately 800 new patients of the National Health Strategy for the Prevention and Control of Sexually Transmitted Infections/HIV-AIDS, offering treatment for over 6,500 patients. 

Interviews were conducted with the head nurse and with the nurses with more years of experience in the service, which served to accurately define the counseling process, know the regulations and norms, identify the duration of interventions, as well as the moments and general characteristics of patients receiving ART and counseling. The proposal of the content began with such description, followed by a discussion with the professionals to identify whether this content reflected the needs and, if not, it was adapted to the reality. Thus, after two or three feedback cycles with some nurses from the team that provided counseling, a final proposal to be submitted to validation was reached.

The second phase comprised the validation of content, construct and clinical validation. The validation was performed by judging experts, focus groups and pilot tests.

Content validity was evaluated by 11 specialists, recognized by their prestige in the area or by reference of nurses and researchers of the service, who were selected for working for over 5 years in the area or for having conducted and published studies on the subject of HIV, alcohol consumption and counseling. The group of 11 specialists consisted of three trained counselors, two of them nurses; one nurse specialist in mental health; two psychologists - one specialist in cognitive behavioral therapy and the other in humanistic therapy; two physicians of the health strategy; two specialists in alcohol consumption; and one educator. 

Content validation evaluated: 1) the clarity of the content, understood as the ease of understanding the message; 2) consistency, determined as coherence in the messages transmitted throughout the document; and 3) congruence, identified as the logical articulation of ideas, parts or segments of the document. An ad-hoc instrument was designed to facilitate the review by the specialist professional of all content present in the Counseling Guide, by segments, following a checklist, and identification of coincidences in the opinion of the judges with regard to each evaluated criterion. Three answer options were provided for each criterion: serves, serves partially and does not serve.

Each specialist was contacted personally, via e-mail and telephone, and received the printed and electronic version of the Counseling Guide to be validated, one instruction manual on the evaluation and the ad-hoc instrument for content validation, the latter via Google Forms. The specialists were granted 12 days to return their evaluations, which were used to improve the Counseling Guide. With an improved version, the following phase was the validation by focus groups.

Participants of the focus groups were seven nurses and 30 patients receiving ART from the National Health Strategy for the Prevention and Control of Sexually Transmitted Infections/HIV-AIDS from the hospital used as the study scenario, from July to December 2017.

The seven nurses worked at the service, and none were on leave or vacation. The patients were older than 18 years, of both sexes, and invited to participate voluntarily in a focus group. Two focus groups of discussion were formed with 15 participants each. To participate in the focus group, patients needed to receive ART in the service, not be in the AIDS phase, not be hospitalized, not suffer mental illness that would limit their will or that would cause dependence on a third individual for taking medications. In each focus group, three relatives or support agents of these patients were also voluntarily involved. 

Two guides were designed for the focus groups, one for nurses and the other for patients, using the “interview guide tool” as the basis - available at the CFIR website^(^ 2 ^)^: http://www.cfirguide.org/guide/app/index.html#/guide_select. The following indicators were evaluated: “perception of key actors on the intervention: whether the perception develops internally or externally”; the “relative advantage”, understood as the “perception of the actors about the advantage of implementing the intervention in the face of an alternative solution”; “adaptability”, as the “degree to which an intervention can be adapted, refined or reinvented to meet local needs”; “reliability”, as the “ability to test small-scale intervention in the organization, and be able to reverse the course (undo the intervention), if necessary”; and “complexity”, defined as the “perceived difficulty of execution, which is reflected in the duration, comprehensiveness, radicality, order, centrality and complexity of the content, and in the number of steps necessary for the application”. The included constructs were defined as follows: the usefulness of counseling, as the “opinion of patients and nurses about the usefulness of the counseling content to improve adherence”; practicality, as “the adjustment of what was discussed in counseling to what happens in reality with patients”; acceptability, as “the participant’s perception that the content proposed in counseling can be voluntarily incorporated into the usual routine of their care”; sustainability, as “the participant’s perception that the proposed counseling can be maintained for a prolonged period without damage to its content or purpose”; and effectiveness, as “the participant’s perception that the proposed counseling can (or with their suggestions) improve adherence to ART of HIV patients who consume alcohol”.

The information expressed in each focus group was recorded with prior authorization given by the participants.

The Guide was improved with the information obtained in the focus groups to move on to the final phase, the clinical validation via a pilot test.

The pilot test was performed with 10 volunteer patients: 5 participated in the initial counseling; and 5 in the follow-up session. The patients who participated in the initial session were those who had recently begun to receive ART, older than 18 years and of both sexes; the patients who participated in the follow-up session were those who had already received some early counseling, volunteers, older than 18 years and of both sexes.

Two nurses were trained to apply the Counseling Guide so the pilot test could be developed. Training sessions were held to clarify any doubts and concerns about the application. 

One nurse applied the Counseling Guide: Initial, that is, for new patients undertaking ART; and the other applied the Counseling Guide: Follow-up. Each of them applied the counseling according to the Guide and evaluated the compliance with the procedure marking YES or NO in a checklist, as well as the difficulties that the process presents, and the start and end time of counseling.

After the application of the pilot test the researchers talked to each of the nurses to identify any difficulties or inconvenience that were not recorded in the instrument.

Considering the results of the pilot phase, the Guide was again improved to its final version. 

For data analysis, the evaluation forms delivered by each specialist were tabulated and the Agreement Index (AI)^(^ 28 ^)^ was evaluated, the binomial test and Aiken’s V coefficient^(^ 29 ^)^ were calculated for each segment of the Guide by evaluated criterion. Validity was considered for agreement index equal to or greater than 80%^(^ 28 ^)^, and p<0.05 significance value for both the binomial test and Aiken’s V coefficient^(^ 29 ^)^. 

Information obtained from the focus groups were individually analyzed for each, considering the evaluated constructs. Following, consensus, agreements and disagreements were identified in the patients’ groups. 

The records obtained from the pilot test were tabulated, and the difficulties in applying the Counseling Guide were analyzed together with the nurses participating in the process. The time employed in each type of counseling was analyzed with measures of central tendency.

The results of the validation were used as feedback to improve the proposed Counseling Guide.

The study was approved by the Institutional Committee for Ethics in Humans (ICEH) of the University (Code 066991) and the Ethics Committee of the Hospital where it was conducted. All participants were guided on the objective of the study, the procedures, risks and benefits, and those who agreed to participate signed an informed consent form. The identity of the participants was protected by anonymity.

## Results

For key actors, i.e., nurses and patients, the source of intervention is internal, despite being externally regulated by the Ministry of Health as the regulatory organization. Regarding the “evidence strength”, counseling shows a IIA evidence level according to the Association of Physicians in AIDS Care Panel, i.e., High evidence: evidence with randomized and controlled trial with important limitations, solid evidence from observational studies, and a strong level of recommendation: “Almost all patients should receive the recommended course of action”^(^ 30 ^)^. 

They following aspects were identified as “relative advantages” of the intervention: the current counseling practice performed in the service but without a formally defined structure; the experience of nursing professionals in counseling; the acceptability of the procedure by patients; the patients’ need for educational and emotional support from the health professionals for adherence; and the recognition of counseling as an intervention performed by nursing professionals. 

Table 1 presents the responses of 8 judges - out of 11 who were invited -, who corroborated in considering that the Guide has a clear, consistent and congruent content. Although the maximum score attributed was two, only in three items up to two specialists attributed the score of one, for this reason, an absolute agreement was not achieved in these segments of the Guide, but rather of majority. In these cases, the content was revised for improvement and suitability.

**Table 1:** Validity of the Counseling Guide according to Experts' Evaluation. National Sanitation Strategy for the Prevention and Control of STI*, HIV/AIDS^†^ of a local hospital. Lima, LI, Peru, 2017

Content	Clarity				Consistency				Congruence			
	AJ^‡^	IA^§^	PB^||^	V^¶^	AJ^‡^	IA^§^	PB^||^	V^¶^	AJ^‡^	IA^§^	PB^||^	V^¶^
Objectives	8	1	0.009	1	6	0.8	0.144	0.875	6	0.8	0.144	0.875
Definitions												
Counseling on adherence to ART^**^ for HIV patients^††^	8	1	0.003	1	8	1	0.003	1	7	0.9	0.027	0.937
Counseling on adherence to ART for HIV patients^††^ who drink alcohol	6	0.8	0.144	0.875	6	0.8	0.144	0.875	6	0.8	0.144	0.875
Counselor profile												
Who develops counseling	8	1	0.003	1	8	1	0.003	1	7	0.9	0.027	0.937
Theories on which counseling is based												
Counseling base to improve adherence to ART** in HIV patients^††^ who drink alcohol	7	0.9	0.027	0.937	6	0.8	0.082	0.875	6	0.8	0.082	0.875
Application of humanistic theory and cognitive-behavioral theory in counseling	7	0.9	0.035	0.937	7	0.9	0.035	0.937	7	0.9	0.035	0.937
Procedure												
How counseling is conducted	5	0.6	0.136	0.75	6	0.8	0.082	0.875	5	0.6	0.136	0.812
Considerations in counseling to improve adherence to ART** in patients who drink alcohol	5	0.6	0.363	0.812	5	0.6	0.363	0.812	5	0.6	0.363	0.812
Initial counseling												
Initial stage	8	1	0.003	1	8	1	0.003	1	7	0.9	0.027	0.937
Central stage	7	0.9	0.035	0.937	8	1	0.003	1	7	0.9	0.035	0.937
ART**	7	0.9	0.035	0.937	6	0.8	0.144	0.875	6	0.8	0.144	0.875
Diet	8	1	0.003	1	6	0.8	0.144	0.875	6	0.8	0.144	0.875
Healthy lifestyle	7	0.9	0.035	0.937	6	0.8	0.144	0.875	6	0.8	0.144	0.875
Adherence to ART**	7	0.9	0.035	0.937	8	1	0.003	1	8	1	0.003	1
Risks of alcohol consumption	8	1	0.003	1	7	0.9	0.035	0.937	7	0.9	0.035	0.937
Final stage	7	0.9	0.035	0.937	6	0.8	0.144	0.875	6	0.8	0.144	0.875
Follow-up counseling												
Initial stage	8	1	0.003	1	8	1	0.003	1	8	1	0.003	1
Central stage	8	1	0.003	1	8	1	0.003	1	8	1	0.003	1
Final stage	8	1	0.003	1	8	1	0.003	1	8	1	0.003	1

* STI = Sexually Transmitted Infections;

† HIV/AIDS = Human Immunodeficiency Virus/Acquired Immunodeficiency syndrome;

‡ AJ = Administrative Judge,

§ IA = Index of Agreement;

|| PB = Poisson's Binomial;

¶ V = Aiken V coefficient;

** ART = Antiretroviral Therapy;

†† HIV = Human Immunodeficiency Virus

In the validation by focal groups, both patients and family members considered the guide of great help to learn more and know what to do about the disease, and they suggested some topics that, according to their experience, require guidance. Similarly, the nurses considered that, although it is possible to apply it, this will take an additional time, but it will ensure the offer of complete counseling for patients to improve their lifestyle and adherence to ART. However, the nurses suggested replacing some technical terms by more common synonyms (Figure 1). The suggested content and recommendations were included in the guide.

**Figure 1:** Assessment of the Counseling Guide by focal groups according to defined constructs. National Sanitation Strategy for the Prevention and Control of STI*, HIV/AIDS^†^ of a local hospital. Lima, LI, Peru, 2017

Constructs	Nurses	Patients and family members
General assessment	Good strategy, useful and important to the patient. Of great help to improve counseling parameters. It allows them to follow steps or procedures regarding the treatment and care that may be offered to the patient. It allows them addressing issues related to reality such as alcohol problems.	It will be of great help to know what to do.
Complexity	The language is adequate.There could be difficulty with drug names. Counseling was very well designed.	Some words are unclear, such as adhesion, so they need to be replaced by more common synonyms.
Utility	It will help patients and their supporters improve or change their lifestyles and improve adherence to antiretroviral therapy. To improve and expand knowledge and quality of life. To detect consumption problems in time and act in a timely manner.	It will help them know the therapy better and how to take it, what to eat and how to take care of themselves. This should include what to do when a dose cannot be taken, how to remedy each situation, the importance of drinking water. It should be started by asking what each patient's lifestyle is like and how the family can show support. To know what harm alcohol does and how to take care of themselves.
Practicality	There could be difficulty to find the time for listening. Nurses could offer counseling. Preparation is recommended.	It could be the time, but if it is important for health, it should be given the necessary time.
Acceptability	Nurses would accept it. It is not considered a waste of time.	It is not considered a waste of time.
Sustainability	It is not a waste of money or resources, it will probably take longer, but it will be helpful.	Yes, it should apply.
Effectiveness	Yes, because it will help improve their knowledge and empowerment. It will help them analyze the situation of those who drink alcohol.	To include what was mentioned.

* STI = Sexually Transmitted Infections;

† HIV/AIDS = Human Immunodeficiency Virus/Acquired Immunodeficiency Syndrome

According to the pilot test, that is, in the application of the counseling guide with patients, we found that the nurse employs 24 minutes, on average, for initial counseling and 21 minutes for the follow-up counseling, with a minimum of 13 minutes. The nurses who participated in the pilot test recognized the benefit of using the guide as to the accuracy, order and complete approach of the content; however, they also identified that the difficulties inherent to the system and the current structure of the service hinder its application, such as the scarce space available to offer counseling, lack of privacy, among others (Figure 2).

**Figure 2:** Assessments of the nurses who participated in the pilot test of the Counseling Guide on the application process and time employed in each type of counseling. National Sanitation Strategy for the Prevention and Control of STI*, HIV/AIDS^†^ of a local hospital. Lima, LI, Peru, 2017

Criteria	Initial counseling	Follow-up counseling
Counselor profile	The participating nurse meets the profile for the counseling process or is in the process for it.	The participating nurse meets the profile for the counseling process or is in the process for it.
Benefits	Allows them to obtain the available information. There is more organization, and counseling is more complete. Patients leave happier.	It allows them to deepen the content. It forces them to complete counseling
Difficulties of the process	They must know how to apply and what instruments should be applied. Lack of privacy during counseling. Confidentiality is needed, space is scarce to offer it properly. There is no comfort, there is overload.	They must know the Guide beforehand; training and simulation are needed. The procedure should be well known. It takes longer, requires deepening. It requires more space and more trained nurses.
Average time taken in minutes ± SD^‡^	24 ± 0.003^‡^	21 ± 0.005^‡^
Maximum time taken in minutes	31	30
Minimum time taken in minutes	20	13

* STI = Sexually Transmitted Infections;

† HIV/AIDS = Human Immunodeficiency Virus/Acquired Immunodeficiency Syndrome;

‡ SD = Standard Deviation

All instruments that are part of the Counseling Guide have a good and high level of reliability (Table 2).

**Table 2:** Reliability coefficient of each of the instruments applied in the pilot test of the Counseling Guide. National Sanitation Strategy for the Prevention and Control of STI*, HIV/AIDS^†^ of a local hospital. Lima, LI, Peru, 2017

Instrument	Cronbach's Alpha	Interpretation
Alcohol Use Disorders Identification Test (AUDIT)	0.65	Good
University of Rhode Island Change Assessment Scale (URICA)		
Precontemplation	0.79	Good
Contemplation	0.94	High
Action	0.93	High
Maintenance	0.88	High
Questionnaire for assessing the adherence to ART^‡^	0.92	High

* STI = Sexually Transmitted Infections;

† HIV/AIDS = Human Immunodeficiency Virus/Acquired Immunodeficiency Syndrome;

‡ ART = Antiretroviral Therapy

The validated Counseling Guide is a structured technical document that aims to “facilitate the practice of health professionals to improve adherence to antiretroviral therapy of HIV patients who drink alcohol.” 

Since the document was designed with a self-instructional and user-friendly format, it starts with the section called “Instructions for use,” where the application scenario, the way of applying, the counseling roadmap, the beneficiaries, and the final recommendations are detailed. 

The objectives (Obj.) of the guide were drafted in a concrete way, consistent with the theme and results-oriented: Theme 1: Antiretroviral Therapy, Obj.: To recognize the antiretroviral treatment and the therapeutic regimen received; Theme 2: Healthy lifestyle, Obj.: To enunciate the characteristics of a healthy lifestyle to improve the adherence to ART; Theme 3: Diet, Obj.: To recognize the existence of different diets and the types of foods that contribute to improving the adherence to ART; Theme 4: Adherence to treatment, Obj.: To explain the adherence to antiretroviral therapy and its importance; Identify the strengths and potentialities to improve and promote adherence to HIV treatment and involve the family or primary caregiver through the mastery of basic knowledge about adherence to ART, if the patient requests it; Theme 5: Alcohol Consumption, Obj.: To enunciate the risks of alcohol consumption, and Theme 6: Strategies to control alcohol consumption, Obj.: To propose strategies to control or reduce alcohol consumption.

In the guide, counseling is defined as “a health intervention strategy performed by trained professionals to generate, in a trust environment, a professional relationship that helps patients identify their health situation, risk factors to which they are exposed, empowers them as to their health management, allows them to develop basic skills to solve problems, and enables them to make decisions and acquire practices that contribute to the adherence to antiretroviral therapy.” And counseling in alcohol-consuming patients is defined as “an oriented counseling for non-dependent alcohol-consuming patients, in order to recognize the risks and consequences of alcohol consumption, develop strategies to avoid, control or reduce consumption at a risk-free level and incorporate healthy behaviors that contribute to improving adherence to antiretroviral therapy.”

In the guide, we propose the counselor profile, which can be easily recognized by the acronym SUCCESS: Sensibility, Utility, Capacity of Listening, Confidentiality, Excellence, Sympathy-Empathy and Soundness. 

The counseling proposal is based on two theories and one model: humanistic T., cognitive-behavioral T. and transtheoretical model.

The humanist approach is applied through the empowerment of the patient for their health care and relationship of trust^(^ 31 ^-^ 32 ^)^. The cognitive-behavioral approach, on the other hand, applies through the learning of favoring conduits, the reinforcement of positive conducts, behavioral activation, among others^(^ 33 ^-^ 34 ^)^.

The transtheoretical model establishes that the change in health goes through six stages: precontemplation, contemplation, preparation, action, maintenance and termination^(^ 35 ^)^. This model applies after identifying the level of alcohol consumption.

**Figure 3:**
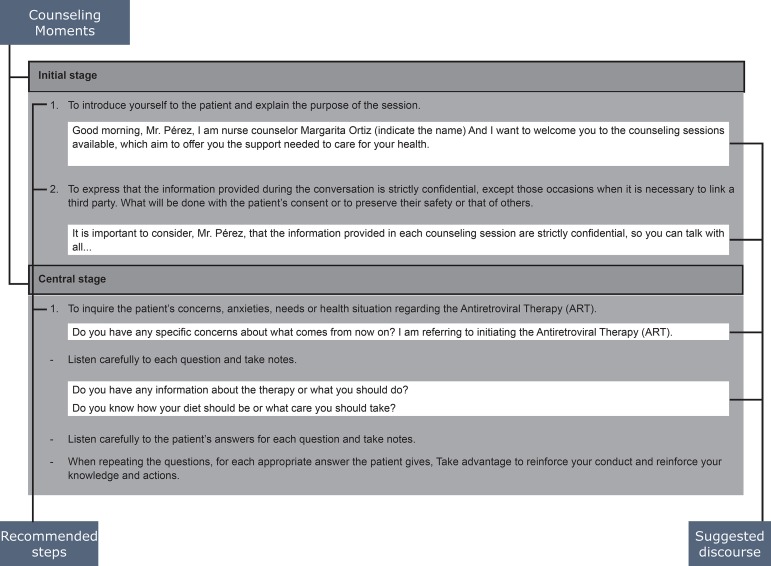
Structure of the Counseling Guide validated to improve adherence to ART* in HIV† patients who drink alcohol. National Sanitation Strategy for the Prevention and Control of STI‡, HIV/AIDS§ of a local hospital. Lima, LI, Peru, 2017 *ART = Antiretroviral Therapy; †HIV = Human Immunodeficiency Virus; ‡STI = Sexually Transmitted Infections; §HIV/AIDS = Human Immunodeficiency Virus/Acquired Immunodeficiency Syndrome

Counseling to improve adherence to ART is still organized in two types of counseling: initial counseling and follow-up counseling. Each of them has three moments: initial stage, central stage, final or closing stage, the Guide includes steps to follow and suggested discourses (Figure 3).

The guide identifies the developing phase, establishes a suggested discourse, and guides the action that the counselor must follow at each moment. 

Initial counseling takes approximately 25 minutes, while follow-up counseling takes 15 minutes, the duration includes the assessment of patient adherence and alcohol consumption.

The guide is complemented with instruments that allow nurses to self-assess in order to identify whether they have the counselor profile, determine the prevalence and level of alcohol consumption of the patient through the Alcohol Use Disorders Identification Test (AUDIT)*,* the predisposition of the patient to change through the “University of Rhode Island Change Assessment Scale,” as well as monitor their level of adherence to ART through the “Simplified Medication Adherence Questionnaire” (SMAQ). Finally, the guide has a form to establish the nursing care plan and two checklists that help the nurse identify whether he/she has reached all stages of the initial and follow-up counseling.

## Discussion

In this article, two important moments are highlighted: the validation process of the counseling guide to improve adherence to ART in patients who drink alcohol and the result of this guide. To rely on the implementation science allowed us to model the guide in a manner consistent with the needs of the service, the patients’ reality and the nursing team. Although the constructs established^(^ 2 ^)^ by the implementation science were analyzed in a gradual and timely manner, each of them contributed significantly to give consistency and solidity with high possibilities of implementing the Intervention in a second phase of experimentation. However, some of the proposed constructs could not be developed because they did not apply to the specific situation or moment, for example, organizational incentives and rewards. Health interventions, programs or policies are designed to promote health or reduce diseases; the assessments should answer specific questions, often related to the configuration, implementation, or results^(^ 36 ^)^.

Content validity in the focus groups and the experts’ opinion helped us identify precise contents and specific guidelines to highlight the usefulness of the guide. The focal group of patients highlighted the need to incorporate useful content for their daily life over which health professionals usually give guidance from a generalized perspective. For example, life in the city requires daily practices such as the habit of eating out, the reduction in leisure spaces or the difficulties to take medication at work hours; what should be considered when adherence to long-term therapies is required. Regarding this, health, housing and safety conditions are known to affect the quality of life of HIV patients^(^ 36 ^-^ 37 ^)^. The inclusion of content to maintain or reduce alcohol consumption at healthy levels was well evaluated by the patients, a theme that was approached in an articulated manner in counseling. A key aspect of the approach of assessment programs or interventions is to identify whether this is appropriate for the target population. Thus, the methodologies of social and mixed research are useful, given that they offer precise evidence of the quality of the intervention and its relevance to the target population^(^ 37 ^)^. 

The experts’ opinion, V level in the ranking of evidence quality according to the practice Model and guidelines based on the nursing evidence of Johns Hopkins^(^ 38 ^)^, allowed us to ensure the quality and consistency of the proposed content and bring a megacognitive look to the document as a self-taught proposal, in order to guarantee the continuity of the counseling process by health professionals. In this sense, we added a section on how to use the guide and how to assess the characteristics of the counselor so that it is not a mere enunciation of attributes, but something that allows their assessment in people interested in providing counseling.

We need to consider that the use of the guide requires training or, at least, a prior reading and complete understanding of the logic of use; before using it in counseling. This will facilitate its proper use and enable the recommended flow according to the patient’s situation. Studies recommend the previous training of counseling professionals to ensure that it is executed according to the configuration, guaranteeing thus its effectiveness^(^ 27 ^)^.

However, the pilot test, the application of mild counseling for approximately 20 minutes, estimated that after acquiring a certain ability in its use, one can be fluid and efficient according to the situation faced by the counselor. One of the attributes of the guide constantly praised by the nurses who rehearsed the application is the scope of its content, which serves as a referent to remember themes relevant to the patient. Regarding this, it is important to highlight that the application of the guide does not limit the freedom of the counselor, since it allows the deepening of some other knowledge or guidance necessary to the patients. Studies indicate that the use of protocols and care guides is necessary and recommended, but that one should not lose sight of the individuality of each patient at the time of intervention^(^ 26 ^)^.

Nowadays, elaborated and validated nursing care and counseling guides are scarce. Usually, these are non-standardized procedures developed at the discretion and experience of the professionals of each service; considering the regulations in force; when it exists. To rely on a validated guide not only evidences a procedure conceived based on scientific knowledge and criteria^(^ 37 ^)^; but it also shows order and systematization of the care offered to the patient or to the target population.

Similarly, given the number of patients that the nurse usually attends in the service and the amount of counseling offered per shift, a validated counseling guide would not only facilitate systematization, but also ensure, to a certain extent, the quality required in care and service. In terms of management, and as a suggestion for future studies, it could guarantee the efficiency and effectiveness of the care offered by the nursing professional.

One of the strongholds of this study was the strong support of scientific evidence for decision-making, from the selection of the intervention to the development of the research process. On the other hand, one of the weaknesses was the limited number of patients who participated in field validation. However, it is important to acknowledge that this phase allowed us to validate the procedure, the times and identify the limitations in the implementation process and, based on that, propose improvements. A research based on an experimental study with a representative sample could provide concrete evidence of the effectiveness of this counseling guide validated in the patient’s adherence to ART.

## Conclusion

The Counseling Guide to improve adherence to ART in HIV-infected patients who drink alcohol has content validity, and its application is considered useful, acceptable, adaptable, sustainable, and potentially effective to improve adherence to the treatment of HIV patients according to their alcohol consumption levels. The validated guide is potentially scalable to other realities in which the profile of patients is compatible: Hispanic adult patients who receive ART and who are not in the AIDS phase; and also, nurses whose training profile and similar language can facilitate the comprehension of its application. Moreover, the method adopted for the validation of nursing interventions, such as counseling in this particular case, has been described in such a way that it can be replicated and adapted to other nursing interventions.
